# INSPEX: Optimize Range Sensors for Environment Perception as a Portable System

**DOI:** 10.3390/s19194350

**Published:** 2019-10-08

**Authors:** Julie Foucault, Suzanne Lesecq, Gabriela Dudnik, Marc Correvon, Rosemary O’Keeffe, Vincenza Di Palma, Marco Passoni, Fabio Quaglia, Laurent Ouvry, Steven Buckley, Jean Herveg, Andrea di Matteo, Tiana Rakotovao, Olivier Debicki, Nicolas Mareau, John Barrett, Susan Rea, Alan McGibney, François Birot, Hugues de Chaumont, Richard Banach, Joseph Razavi, Cian Ó’Murchú

**Affiliations:** 1 University Grenoble Alpes, CEA, LETI, F-38000 Grenoble, France; 2 CSEM SA, 2002 Neuchâtel, Switzerland; 3 Tyndall National Institute, T12 R5CP Cork, Ireland; 4 STMicroelectronics S.r.l, 80022 Arzano (Naples), Italy; 5 OnSemi, Airport Business Park, T12 CDF7 Cork, Ireland; 6 University of Namur, CRIDS, 5000 Namur, Belgium; 7 Cork Institute of Technology, Bishoptown, T12 P928 Cork, Ireland; 8 GoSense, 38 rue de l’Université, 69007 Lyon, France; 9 School of Computer Science, University of Manchester, Manchester M13 9PL, UK

**Keywords:** ultrasound, LiDAR, ultra-wideband radar, environment perception, data fusion, portable device, smart system, visually impaired and blind (VIB), wearable, portable

## Abstract

Environment perception is crucial for the safe navigation of vehicles and robots to detect obstacles in their surroundings. It is also of paramount interest for navigation of human beings in reduced visibility conditions. Obstacle avoidance systems typically combine multiple sensing technologies (i.e., LiDAR, radar, ultrasound and visual) to detect various types of obstacles under different lighting and weather conditions, with the drawbacks of a given technology being offset by others. These systems require powerful computational capability to fuse the mass of data, which limits their use to high-end vehicles and robots. INSPEX delivers a low-power, small-size and lightweight environment perception system that is compatible with portable and/or wearable applications. This requires miniaturizing and optimizing existing range sensors of different technologies to meet the user’s requirements in terms of obstacle detection capabilities. These sensors consist of a LiDAR, a time-of-flight sensor, an ultrasound and an ultra-wideband radar with measurement ranges respectively of 10 m, 4 m, 2 m and 10 m. Integration of a data fusion technique is also required to build a model of the user’s surroundings and provide feedback about the localization of harmful obstacles. As primary demonstrator, the INSPEX device will be fixed on a white cane.

## 1. Introduction

Obstacle avoidance systems for autonomous vehicles fuse multiple range sensing technologies (i.e., LiDAR, radar and ultrasound) and visual to detect different types of obstacles over various lighting and weather conditions. Range sensor data are combined with the vehicle orientation (obtained for instance from an inertial measurement unit and/or compass) and navigation subsystems. These obstacle avoidance systems are power hungry and require powerful computational capability, which limits their use to high-end vehicles and robots.

INSPEX, which stands for “INtegrated smart SPatial Exploration system”, is a H2020 project that intends to make obstacle detection capabilities available as a personal portable and/or wearable multi-sensors miniaturized low power device. Thanks to the different range sensing technologies embedded (i.e., ultrasound, LiDAR, depth camera, ultra-wideband radar), the INSPEX device detects, localizes and warns about obstacles under various environmental conditions, in indoor/outdoor environments, with static and mobile obstacles. Applications of the device comprise safer human navigation in reduced visibility conditions (e.g., for first responders and fire brigades), small robot/drone obstacle avoidance systems and navigation for the visually and mobility impaired people.

**Figure 1:**
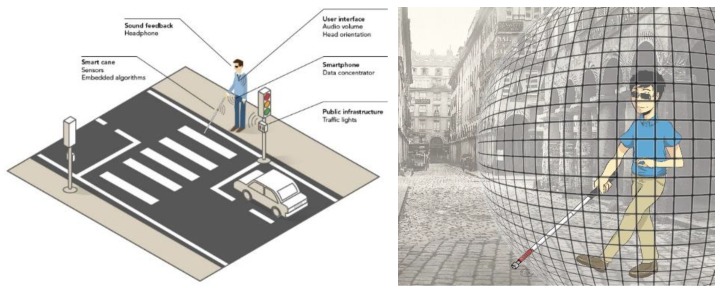
**Left**: primary demonstrator. **Right**: safety cocoon offered by the INSPEX system.

As primary demonstrator, we will plug the INSPEX device on a white cane for Visually Impaired and Blind people (referred as VIB people in the paper). With a slight modification of the arrangement of the sensors, it could become a wearable device attached on the person. The INSPEX device will detect obstacles over the whole person height and provide audio feedback about harmful obstacles in the user’s environment. In this way, it will improve the user’s mobility confidence and reduce injuries, especially at waist and head levels [1]. The device will offer a “safety cocoon” to its user: it will provide feedback only when obstacles enter the safety zone. It will limit the cognitive load [2] that is usually observed with standard smart white canes that provide feedback as soon as they detect an obstacle. Figure 1 summarizes the INSPEX project primary demonstrator (left). It also illustrates the “safety cocoon” concept (right).

The primary goal of INSPEX is to optimize the range sensors embedded in a portable device that detects obstacles in its surroundings. To provide realistic constraints, the smart white cane application was chosen. Therefore, the range sensors must be optimized in size, weight and power consumption to make them compliant with the system requirement derived from the user’s needs and expectation expressed in terms of size, weight and lifespan between two battery recharges. This encapsulates the main results presented in the present paper.

The rest of the paper is organized as follows. Section 2 shortly reviews the related work. Then, Section 3 deals with INSPEX methodology. It summarizes user’s needs and the main system requirements, in particular regarding the characteristics of the different range sensors. A subsection is dedicated on the compliance to the General Data Protection Regulation (GDPR) [3]. Section 4 shows the optimization achieved in the course of the project for each range sensor. Section 5 shows how the fusion of measurements from different range sensors enriches the knowledge about obstacles and the free-space in front of the device. Section 6 summarizes the main achievements from a sensor perspective and provides future work directions. 

## 2. Related Work

Among the different aids to help VIB people in their daily commute, the most accepted and largely used one still seems to be the traditional white cane, even if it is not sufficient to for a safe mobility of its user. Indeed, it does not detect obstacles at waist, chest or head level. To answer the traditional white cane limitations, electronic white canes have been developed in the last decades and some solutions already exist on the market [4]. Moreover, many research projects address these systems from various point-of-views, ranging from the sensing aspects to the user’s feedback and warning, but also embedded computing needs that depend on the sensing technologies. D. Dakopoulos and G. Bourbakis [5] define *Electronic Travel Aids* (ETA) as “devices that transform information about the environment that would normally be relayed through vision into a form that can be conveyed through another sensory modality. They show that ETA are categorized depending on how the information is gathered from the environment and how it is given back to the user (sound, vibration). They also review 22 Electronic Travel Aids, 5 of them being already products at the time the paper was published (2010). Some of these solutions have nothing to do with a white cane: glasses, helmets and belts have also been proposed in the literature.

Thanks to progress in microelectronics and computer science, new solutions have been explored or are under development. This strong involvement of the scientific community is closely connected with enhancing the mobility of VIB people. In fact, according to the World Health Organization observation in 2018 [6], “1.3 billion people live with some form of vision impairment. With regards to distance vision, 188.5 million people have mild vision impairment, 217 million have moderate to severe vision impairment, and 36 million people are blind” [7].

Regarding perception of the environment, all sensors that are able to sense obstacles have been implemented, together with appropriate Signal Processing. Ultrasounds are widely used [5,8]. because of their low cost but their operating range is limited due to problems when operating with highly reflective surfaces (e.g., smooth surfaces), with low incidence angle of the beam and when detecting small openings (e.g., a narrow door) [9]. Infrared sensors are also reported in the literature [10,11] even if they are seldom used. Optical and vision systems [12] seem appealing because they can offer obstacle recognition functionality when associated to AI techniques [13]. Unfortunately, they suffer from a high sensitivity to the natural ambient light. To improve their performance, they can be associated with another sensing modality [14]. Electromagnetic sensors seem a viable solution and several ETA that embed radar have been proposed recently, see [9] and references therein. They do not suffer from the drawbacks of other sensing modalities. However, they require heavy signal processing to extract useful information regarding the localization of the obstacle in the sensor field-of-view. Moreover, their ability to detect an obstacle depends on the obstacle itself (e.g., size, shape, material, incidence angle).

Co-integration of several range sensing modalities seems a valid option to overcome the drawbacks exhibited by each of sensor technology and improve the overall device detection capabilities. A few solutions exist in the literature. For instance, [14] implements ultrasound sensors together with vision based techniques to detect and recognize obstacles. INSPEX consortium adopted this multi-modality sensing approach: ultrasound, LiDAR, depth camera (time-of-flight) and radar sensors are embedded in an integrated device together with an IMU and computational capabilities from the consumer market. A model of the user’s environment is built via the fusion of the range measurements using SigmaFusion™ [15]. The environment is in the form of an occupancy grid [16]. This latter is composed of disjoint cells, each one bearing a probability of occupancy in [0; 1] reflecting its likelihood of being occupied by an obstacle. SigmaFusion implements a Bayesian fusion technique in a fully revisited algorithm that performs computations using integer arithmetic only, making it suitable for embedded devices.

## 3. INSPEX Methodology 

### 3.1. Overview

The design of a novel support for disable people must truly answer user’s needs and expectation to ensure its acceptance and adoption. This requires an effective and ongoing dialogue between the VIB community and the various professionals (incl. researchers and engineers) that develop and provide ETA [17]. At the beginning of the project, the INSPEX consortium conducted interviews in different European countries to collect users’ needs [18] requirements and expectation regarding the development of a new ETA. Even if some of them would like a hand-free (wearable) device for specific activities, most of interviewees prefer to *keep the traditional white cane*, and expand it with new functionalities. Not surprisingly, and in accordance with other studies [17], end-users request a *non-stigmatizing*, as much as possible unnoticeable and *easy to carry* device. This later aspect is of high importance because of harm fatigue induced by a heavy device. They also expressed needs regarding *early warning* (in the range of a few seconds) of nearby obstacles that they may collide with to slightly and smoothly modify their trajectory. Taking into account the mean walking speed, this corresponds to a detection range of a few meters. They also expressed difficulties in detecting particular obstacles, for instance, parking barrier or bistro table at pelvis level, road sign at waist or head level.

**Figure 2:**
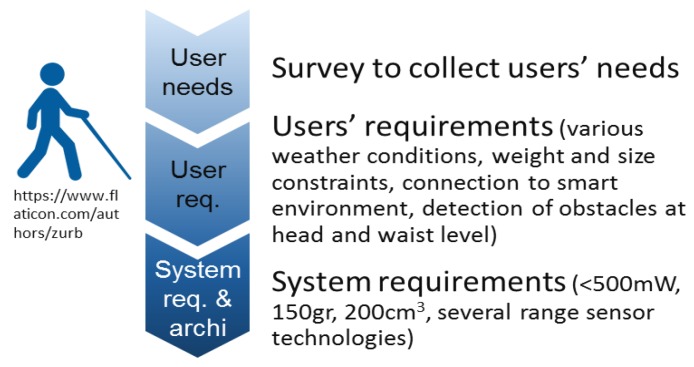
INSPEX methodology.

We gathered the needs collected in six *Personas* that are fictional characters, representing various profiles of the targeted end-user community [19]. These personas can be deeply described and analyzed without any concern about privacy. From these Personas, we derived *functional and non-functional requirements* together with their priority of implementation in the integrated prototypes that are developed in the course of the INSPEX project. Note that the end-users’ requests advocate for pushing constraints on the device integration aspects (especially size and weight) even further. This involves a strong optimization of the sensor prototypes brought to the project by partners in power consumption (to decrease the size of the battery) and in size to ease the system integration and decrease its weight. Then, we derived the *system requirements* for the VIB application, and finally we proposed the system architecture [20]. Figure 2 illustrates this methodology.

**Figure 3:**
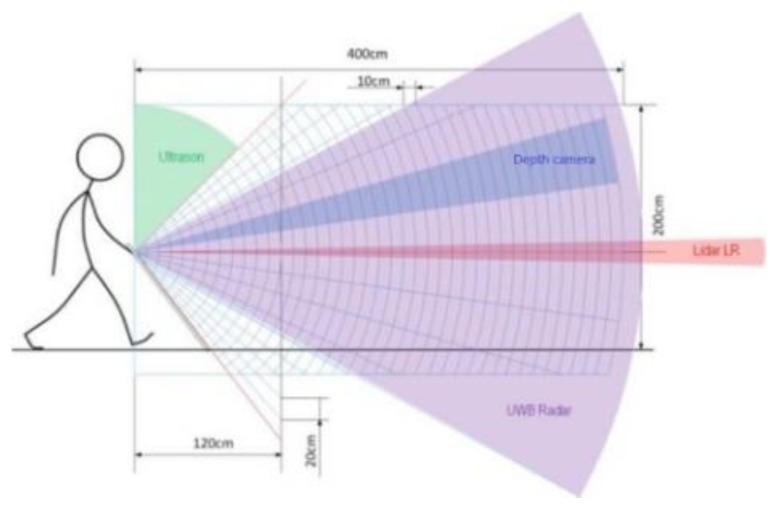
Arrangement of the sensors to cover the whole person’s height.

To ensure the proper detection of different obstacles (in size, shape and material) in various environmental conditions (including fog, snow, rain, direct sunlight) over the whole person’s height, we implemented four range-sensing technologies in the INSPEX device, namely, ultrasound, LiDAR, depth camera (time-of-flight) and radar. Figure 3 shows how these sensors are arranged to detect obstacles over the whole person height. As can be seen, the different fields-of-view overlap to offset drawbacks of one technology by another one. For instance, the “long-range LiDAR” is able to detect obstacles far from the user thanks to its large measurement range. It will efficiently detect obstacles a pelvis level (e.g., parking barrier or bistro table) because of its mechanical arrangement. However, due do its narrow field-of-view, it will miss obstacles located at foot or waist levels. Moreover, in case of bright direct sunlight, it may provide unreliable measurement. Lastly, it will miss glass windows or glass doors. The ultra-wideband radar, even with its smaller measurement range, provides complementary information that offsets drawbacks of the long-range LiDAR. In addition, it can warns the user in advance about obstacles at head level, as well as the ultrasound that aims upward and detects nearby head-level obstacles.

Table 1 summarizes the main system requirements from a sensing perspective as derived by the partners from the users’ requirements and from the optimization of the sensors envisioned in the course of the project. Note that the numerical values come from a breakdown of the weight, size and detection range for the integrated device as expressed by the potential users. The power budget assigned to each sensor comes from the device lifespan between two battery charges and from the battery acceptable weight.

### 3.2. INSPEX and Its Legal Compliance to the GDPR

The INSPEX device delivers to the user information about obstacles entering the “safety cocoon” through his/her smartphone. Therefore, the consortium had to deal with the device’s compliance with the GDPR (EU General Data Protection Regulation) [3].

This latter required first to identify the processing of personal data that are likely to fall under the territorial and material scope of data protection rules. This lead to take into consideration the data flows between the INSPEX device and the mobile application loaded on the user’s smartphone, as well as the data flow generated when using the mobile application.

The second step of the legal analysis consisted in identifying the actors (data controller, data processor and all the other recipients) involved in the delivery of a service, for instance, a geolocalization one. Other recipients refer for instance to the situation where the coordinates of the user are sent with the smartphone IP address to the provider of a geolocalization service.

Then, the legal analysis focused on the rules applicable to the processing of personal data in this context. This refers to the seven principles governing the processing of personal data, namely, lawfulness, fairness and transparency, purpose limitation, data minimization, accuracy, storage limitation, integrity and confidentiality, and accountability. It also refers to the general obligations of data controller and processor (e.g., privacy by design and by default), to the security of personal data, the necessity to perform a data protection impact assessment, without forgetting to implement appropriate mechanisms and procedures to ensure the effectiveness of the data subject’s rights when using the INSPEX device.

## 4. Optimization of Sensors and Their Results

Partners brought to the project: prototypes of an ultrasound, a long-range LiDAR, a depth camera (time-of-flight) and an ultra-wideband radar.

We first characterized these modules using a common methodology: typical obstacles (e.g., bistro table, tree branch) were placed at predefined distances in from of the prototypes in controlled environmental conditions. Tests in mobility were also conducted with the ultra-wideband radar.

Then, optimization of the prototypes was carried out to make them compliant with their requirements in terms of measurement range, size, weight, and power consumption, see Table 1.

This optimization has been conducted by the prototype owner and it depends on the characteristic that must be improved. Section 4.1, Section 4.2, Section 4.3, Section 4.4 present for each sensor module, the prototype brought to the project, its optimized version and some characterization results after optimization.

Note that the optimization phase has delivered a preliminary version and a final version of the optimized modules. Only results for the final version are provided here. INSPEX partners also chose to develop a stand-alone version of each module even if this increases the consumption, size and weight of the overall integrated device. In this way, each module can extend its potential exploitation routes. Lastly, the project delivers prototypes of the optimized sensor modules at TRL4 [21]. Therefore, their cost is not considered in the present work, even if it will have a strong impact on the market uptake of their more mature version.

### 4.1. Ultrasound Module

The ultrasound module must be designed to detect obstacles up to 1 m from the user, see Table 1. The prototype brought to the project is compliant with its requirements in terms of range but its power consumption is too high and its size and weight must be slightly decreased.

#### 4.1.1. Ultrasound Prototype

**Figure 4:**
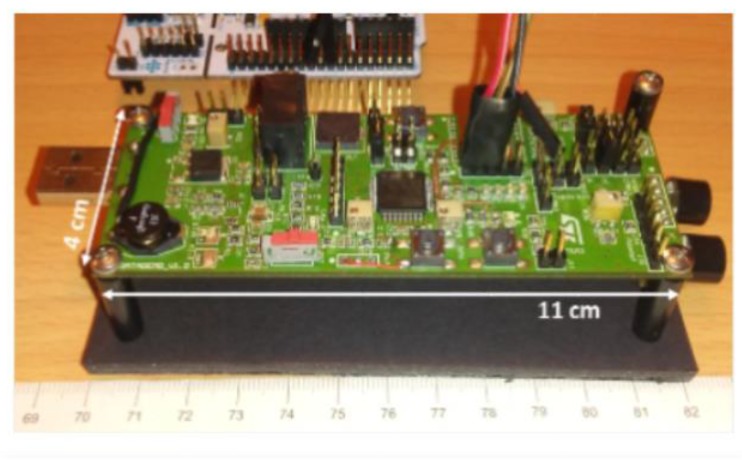
Ultrasound prototype module.

The prototype brought to the project is depicted on Figure 4. Its weight is 44 g with a dimension of 110 × 40 × 30 mm. The input power is equal to 400 mW. Its measurement range is less than 1 m, which is not compliant with its requirements. Its field-of-view is larger than 25°, which is sufficient to cover the head level, see Figure 3. 

#### 4.1.2. Optimized Module

The optimization activities addressed mainly the following aspects:

- Size reduction: the board initial area was halved by careful layout and component selection. The transducers were changed to smaller ones (MA40 HIS from Murata);
- Power consumption: the whole bill of material was revised, in particular the power management section and the microcontroller. Moreover, the firmware was optimized to switch on the different subcircuits only when needed;
- The software algorithm for obstacle detection was changed from threshold-based to cross-correlation-based, and optimized to run as fast as possible on the microcontroller in real-time.

**Figure 5:**
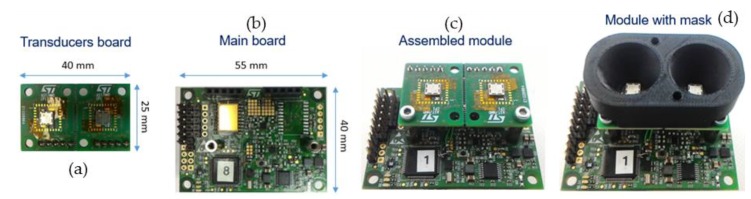
Ultrasound module components: (**a**) transducer board; (**b**) main board; (**c**) ultrasound module; (**d**) ultrasound module with cone-shaped mask.

The ultrasound module is composed of a main board, with the driving electronic, and a transducer board, mounted on top of the main board, see Figure 5. The module features up to four transducers, up to four ASICs, each one capable to drive one transmitter and read one receiver, an optional additional analog front-end with all-pass filters, a temperature/humidity sensor to compensate the speed of sound, a microcontroller from STM32 L4 family, and a power management section. On top of the transducer board, a cone-shaped part may be mounted to increase the directivity and sensitivity.

To measure the distance with respect to a target, we developed an algorithm based on cross-correlation between the received signal and a fixed reference echo [22]. The algorithm runs in real-time on the microcontroller at up to 25 measures per second (2-meter range) or 10 measures per second (5-meter range).

#### 4.1.3. Results for the Optimized Ultrasound Module

The optimized module characteristics are reported in Table 2 together with the prototype and requirement ones. As can be seen, the optimized ultrasound module is compliant with its requirements with no change in the field-of-view (≈25°). The power consumption can be decreased via time triggering techniques.

**Figure 6:**
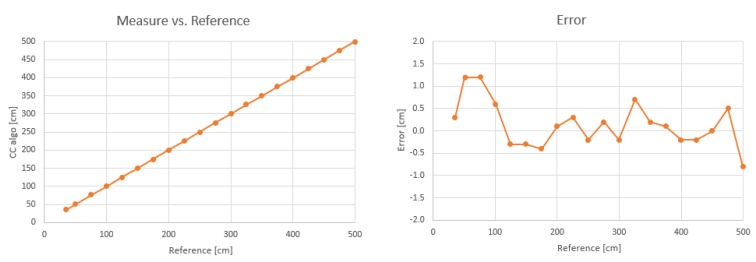
Measured distance vs. reference and its error.

The measurement accuracy was characterized by placing the module in front of a wall, at known distances. The reference distance was measured with a tape measure. Two transducers were used, one for transmission and one for reception. Figure 6 shows the distance measurement error, with single measurements (no averaging) and no further compensation: the error has a mean of 0.14 cm and standard deviation of 0.51 cm, in a range from 35 cm up to 5 m.

In order to reach the 5-meter range the cone-shaped mask must be mounted, otherwise, the module still works, but with its range reduced to 2 m. The cone-shaped mask has also the effect to reduce the field-of-view (defined as −6 dB decrease of the sound pressure level [SPL]), from ± 80° without the mask, to ± 20° with the mask, in both elevation and azimuth.

The capability to detect different types of obstacles was characterized by placing obstacles of different shapes and materials at the distance of 1 m from the sensor and measuring the intensity of the received echo. Results are shown in Table 3, where the intensity is in arbitrary unit, proportional to the SPL at receiver.

### 4.2. Long-Range LiDAR

The long-range LiDAR module must be designed to detect obstacles up to 10 m from the user. The prototype brought to the project is compliant in terms of range (25 m) but its components were not optimized for weight (465 g), size (119 × 66 × 77 mm^3^) or power consumption (1.32 W).

#### 4.2.1. Long-Range LiDAR Prototype

**Figure 7:**
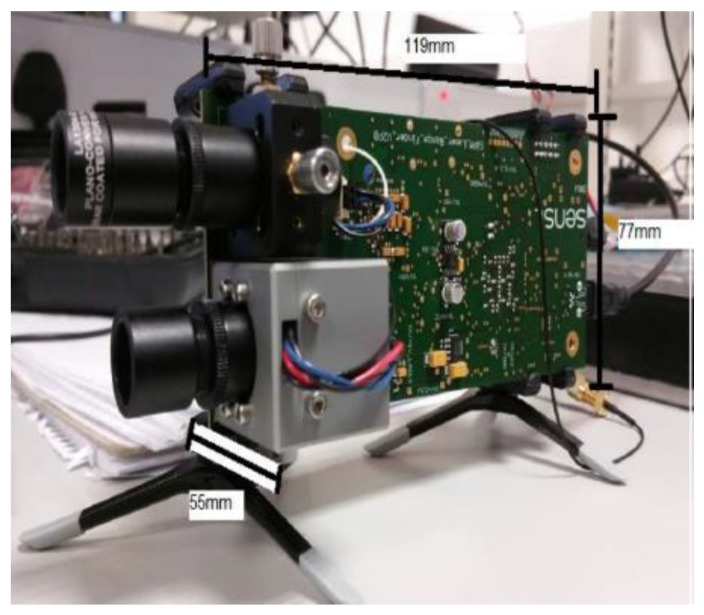
Long-range LiDAR prototype brought to the project.

OnSemi designed this prototype. It comprises a single board with a pulsed laser diode and collimation lens and an OnSemi photomultiplier with focusing lens. The focusing lens is coated to reduce interference from light outside of the 905 nm wavelength. The board also contains an FPGA for control of the detector and data processing. The board is powered through the mains and communication is achieved through a specific computer program. The prototype is depicted on Figure 7.

#### 4.2.2. Optimized Module

As the range required is not as large as the original prototype, it was possible to reduce the power consumption. This involved a redesign of the motherboard pre-amplification circuit. The size of the optics was also substantially reduced compared with the original implementation.

**Figure 8:**
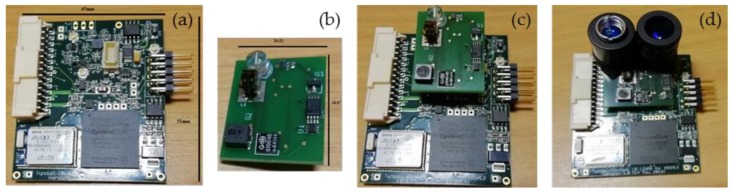
Optimized long-range LiDAR Components: (**a**) Motherboard; (**b**) Optics board; (**c**) Module; (**d**) Module with Optics.

The optimized module comprises a motherboard and an optics board to allow for different configurations when integrated into the INSPEX device, see Figure 8. The motherboard contains a microcontroller and an FPGA to drive the optics devices and to process the data and provide power management. The optics board includes the laser diode and the silicon photomultiplier to detect the returned laser signal. The lenses for focusing and collimating the beam can be added to the board (Figure 8d) or integrated into the package. When working in stand-alone, communication is achieved through a specially designed computer program. An additional Bluetooth module allows data collection on a smartphone app.

#### 4.2.3. Results for the Optimized Long-Range LiDAR Module

The optimized module characteristics (design and measured) are reported in Table 4 together with the prototype and requirement ones. We decreased the power consumption but not enough to meet the module requirements. One solution is to implement duty cycling in the power manager of the INSPEX device to decrease the mean power consumption. The other characteristics are consistent with the requirements.

To evaluate the measurement, a single point measurement of a stationary object at a known distance is performed. The laser diode (SPL PL90) is pulsed at 150 ps. A clock is started at the initiation of the pulse. The detector is a MicroFC-10020. When a returned signal is detected, the clock is stopped, the time-of-flight is stored and the distance is computed on the FPGA. The bias is computed after the initial measurement and the measurement frequency can be decreased to reduce the power consumption without a significant decrease in accuracy. 

### 4.3. Depth Camera

The depth camera (time-of-flight) module must be designed to detect obstacles within a volume of 4.0 m length, 2.0 m height and 1 m width (0.5 m on each side of the user), in front of the user. The module should be able to detect obstacles as small as 0.1 m x 0.1 m at a user speed of about 5 km/h. It should measure reliably at day and night, and under different weather conditions, e.g., cloudy, foggy, rainy, and snowy. Moreover, it should be as tolerant as possible to sunlight.

#### 4.3.1. Depth Camera Prototype

As prototype, we chose the off-the-shelf evaluation kit epc635 from ESPROS Photonics Corporation [23]. It is a fully assembled and tested camera system designed for the evaluation of the epc635 time-of-flight imager with a resolution of 160 × 60 pixels that can achieve a frame rate of up to 128fps with an absolute accuracy in the centimeter range after calibration and runtime compensation [24]. While the long-range LiDAR delivers a unique measurement (i.e., a unique beam), the epc635 imager produces a point cloud that covers a wider area space with a field-of-view of 56°.

Preliminary measurements with the evaluation kit showed that the imager characteristics are compliant with the depth camera requirements. They are not reported in the paper.

#### 4.3.2. Optimized Module

**Figure 9:**
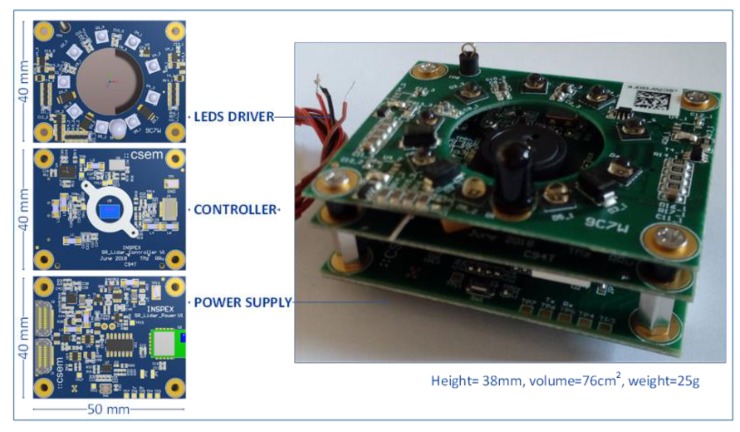
Depth camera (time-of-flight) module.

The depth camera module has been designed “around” the epc635 time-of-flight imager as a stack of three PCBs split by function, i.e., power supply, controller and LEDs driver (see Figure 9). The optical part is based on a newly designed holder and commercial components, the whole module being customized and optimized for the INSPEX application.

#### 4.3.3. Results for the Optimized Depth Camera Module

The optimized module characteristics are reported in Table 5 together with the prototype and requirement ones. The power consumption slightly exceeds the module requirements and duty cycling could be used to meet (in mean value) the requirements. The other characteristics are consistent with the requirements, with a slightly better measurement range. The measured field-of-view is equal to 33.5° in elevation and 40° in azimuth.

**Figure 10:**
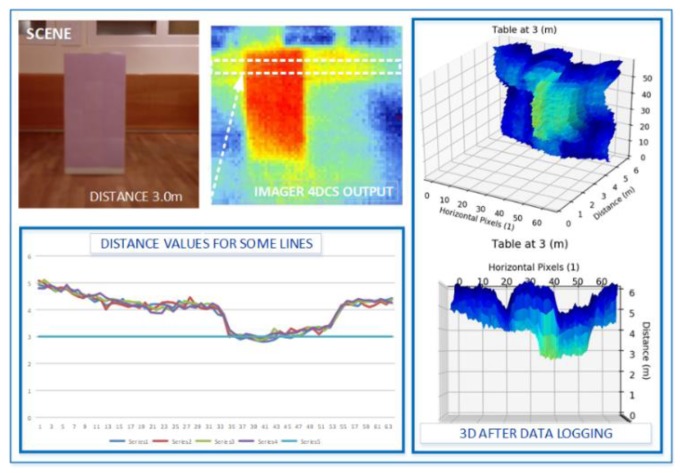
The depth camera (time-of-flight) module can detect objects located at a distance up to 4 m under controlled conditions.

Figure 10 illustrates what the depth camera module “sees” when an obstacle is placed at 3 m in a controlled environment. This result is obtained with calibration parameters that are stored in the module. More tests are currently performed to fine-tune these parameters. Basically, the depth camera (time-of-flight) module produces raw distance and amplitude values. Runtime compensation and dynamic adjustment must be integrated in the module to work as expected under different environmental conditions.

### 4.4. Ultra-Wideband Radar

The ultra-wideband (UWB) radar module must be designed to detect obstacles up to 4 m from the user. The prototype brought to the project was designed to operate at 4 GHz for breath monitoring in intensive care units. Therefore, its characteristics are far from the module requirements: its size is 9 × 9 × 3.5 cm^3^, it weighs 200 g, and it has a power consumption of 825 mW.

#### 4.4.1. Ultra-Wideband Radar Prototype

**Figure 11:**
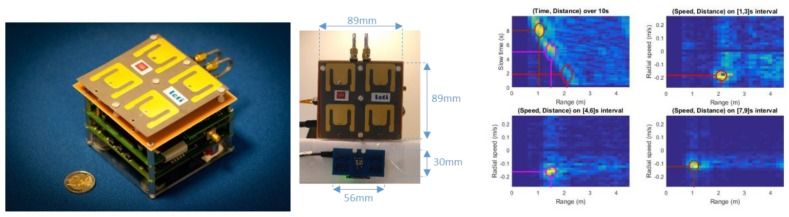
Ultra-wideband radar prototype (**left**, **middle**) and its response over time (3 snapshots) (**right**).

The prototype is depicted on Figure 11. It is made of a RF board connected to an antenna board, a digital board connected to the RF board on the one side and to an external microcontroller board through Serial Peripheral Interface (SPI) on the other side. The external microcontroller board is a Nucleo 144 board (with a STM32F7 microcontroller). Lastly, a software driver interfaces the external microcontroller board with a PC over an Ethernet physical interface and Transmission Control Protocol/Internet Protocol (TCP/IP) and User Datagram Protocol/Internet Protocol (UDP/IP).

After a static characterization phase of the UWB radar, we performed measurements in mobility conditions. The radar is placed on a cart moving linearly towards the obstacle with a colliding trajectory. The relative radial speed is about −0.15 m/s. The right drawing in Figure 11 shows the radar response over time with three snapshots in the speed domain. The top left curve clearly shows the “approaching” obstacle wavefront while the three other curves show how the speed can be exploited in the estimation of colliding trajectories.

#### 4.4.2. Optimized Module

**Figure 12:**
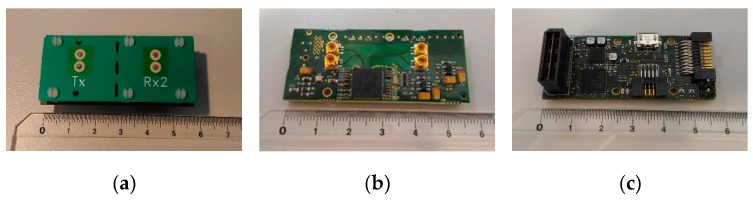
Ultra-wideband radar module. (**a**) Antenna board; (**b**) RF board; (**c**) digital board.

The ultra-wideband radar module is mainly an optimization in form factor and integration of the initial prototype. It is made of three stacked boards, namely, the antenna board, the RF board and the digital board, see Figure 12. The digital board has the FPGA and the SPI interface to the INSPEX main computing platform. The module main characteristics are:

- 8 GHz operation (instead of 4 GHz for the prototype);
- Antenna: Azimuth beam 56° (3 dB), 118° (10 dB), Elevation beam 56° (3 dB), 118° (10 dB), linear, vertical polarization;
- Up to 10 m range, 15 cm resolution, 200 Hz raw data refresh rate (acquisition rate);
- Non-ambiguous relative speed estimation over [−1.875, 1.875] m/s;
- Raw data baseband (I, Q) signal over the 64 distance bins (fast “channel” time axis);
- SPI interface to the general processing platform embedded in the overall device;
- Low-level signal Processing performed on the INSPEX main computing platform.

#### 4.4.3. Results

The optimized module characteristics are reported in Table 6 together with the prototype and requirement ones. The power consumption exceeds the module requirements. However, this value corresponds to the peak (measured) one. The field-of-view is equal to 56° in elevation and 56° in azimuth (3-dB beam width), which is consistent with the requirements. The main underperformance is the measurement range that is clearly less than expected.

**Figure 13:**
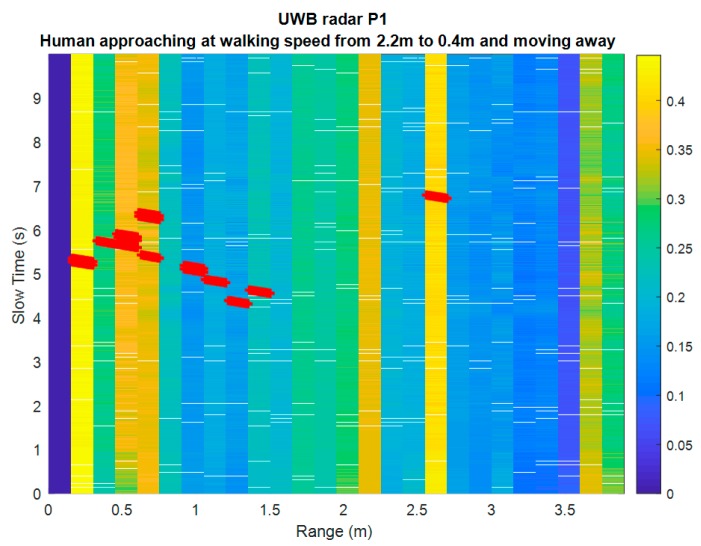
Example of performance verification with a human walking towards the radar.

Figure 13 shows a test result where the obstacle is a human person approaching the radar at a walking speed from 2.2 m at time 3.5 s and moving away from 0.4 m at the same speed at time 6 s. This example result can be straightforward compared to the characterization results of the prototype for which a walking person was successfully detected up to 8 m away.

The reduction of range between the prototype operating at 4 GHz and this optimized module operating at 8 GHz is therefore in the order of 30 dB, which is explained as follows:

- The effective isotropic radiated power (EIRP) output power is reduced by 8 dB (~6 dB for the integrated circuit itself and ~2 dB for the antenna with a larger elevation beam);
- The path loss is increased by 6 dB for the same distance since the frequency is doubled;
- The receiver antenna gain is reduced by 2 dB as for the transmitter one;
- The receiver noise is degraded by about 9 dB, which was measured for the ultra-wideband integrated circuit.

In total, the estimated degradation of the optimized module versus the prototype in terms of received signal to noise ratio, independently of the capability of the receiver static clutter rejection, is about 25 dB, which coincides with the human walking test case degradation. As a human body has about 1 m² RCS and obstacles as small as 0.01 m² RCS shall be detected up to a few meters. The range measurement of the optimized module cannot meet the Table 5 requirements, even with further optimization.

## 5. Data Fusion to Build a Model of the Environment

Data acquired by the different range sensors are fused in real-time using the SigmaFusion™ technology [15] that builds an occupancy grid to model the device surroundings.

An occupancy grid, initially introduced by [16], is composed of a finite number of disjoint cells. A probabilistic estimate of the possible state is assigned to each cell. A cell can have two possible states: “occupied” or “empty”. It is considered occupied if an obstacle is present, partially or totally, in the cell. It is considered empty otherwise. An obstacle can be any possible body in the scene, for example cars, human beings, animals, plants, buildings, traffic signs, etc. The occupancy state of each cell is estimated through a perception model evaluated for each measurement returned by the range sensors. Since every sensor embodies uncertainties and errors, the estimation of each cell state incorporates uncertainties. This uncertainty is represented through a probability of occupancy assigned to each cell, hence the name occupancy grid. Bayesian fusion is applied to fuse measurements from different range sensors. Note that even if this fusion process exhibits exponential complexity in its general formulation, the “nearest target” hypothesis allows breaking down this complexity to a linear one. This hypothesis means that we consider in the fusion process the smallest distance to an obstacle returned by a sensor, the information the measurement might provide behind this nearest obstacle is not taken into account to build the environment model.

SigmaFusion™ revisited the occupancy grid algorithm to manipulate only integer numbers instead of floating point ones, leading to the possibility to implement the Bayesian fusion algorithm on a microcontroller. This change in the arithmetic paradigm makes occupancy grid computation implementable on consumer market microcontroller instead of high-end processors.

**Figure 14:**
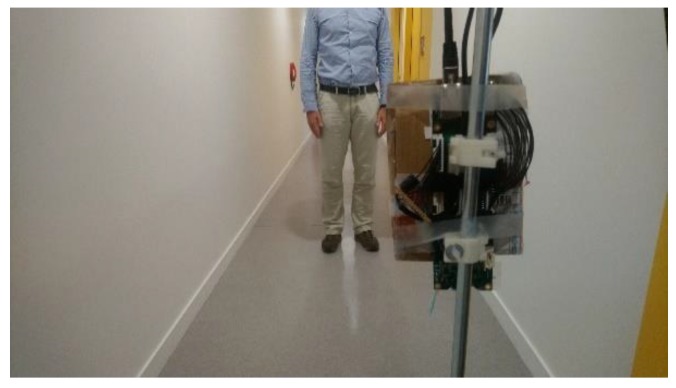
Experiment setup: the preliminary INSPEX prototype is fixed on a white cane; the device tries to detect obstacles (walls, objects, people) in a corridor.

Even if this paper is not centered on data fusion, we now show how fusion of data acquired for different range sensing technologies improves the environment modeling. Figure 14 shows the experiment setup. The preliminary INSPEX prototype is fixed on a white cane. It contains the ultrasound sensor, the depth camera and the General Processing Platform on which data fusion is performed using SigmaFusion™. Data are also stored to plot in 3D the scene that the sensors “see” and how it is modeled with the fusion algorithm.

**Figure 15:**
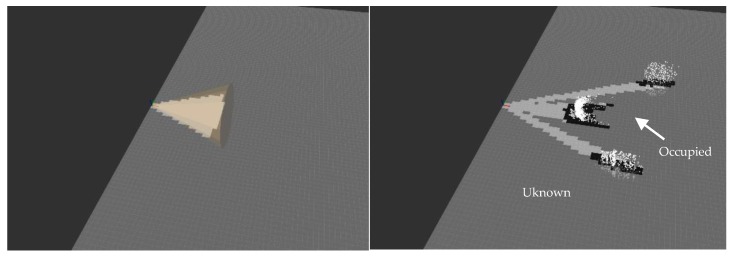
Ultrasound (**left**) and depth camera (**right**) measurements together with their respective occupancy grid computed with SigmaFusion™. The color of each cell encodes the occupancy probability (black: “occupied”; light grey: “empty”, grey: “unknown”).

Figure 15 shows measurements of the ultrasound sensor (left) and of the depth camera (right) plotted in 3D. The first one presents a cone-shape while the second one is a point cloud. The occupancy grid computed with SigmaFusion™ from each sensor data is superposed on the measurement plots. The cells in dark grey (light grey) encode a high (low) probability of occupancy while the cells in grey correspond to the situation where the probability of occupancy is equal to 0.5, which means that we do not know if the cell is empty or occupied. Note that the occupancy probability ranges over its full span, ranging from 0 (empty–encoded in white on the plots) to 1 (occupied–conceded in black on the plots).

**Figure 16:**
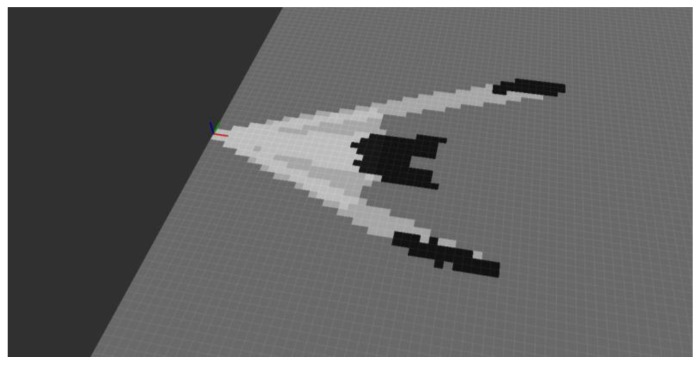
Occupancy grid computed from both the ultrasound and the depth camera measurements.

As can be seen, the occupancy grid computed from the depth camera measurements does not provide information regarding the presence/absence of obstacles in some areas, typically, the ones where no obstacle is found up to the maximum sensor range (for the actual experimental setup in Figure 14, between the person and the walls). The occupancy grid computed from the ultrasound and the depth camera measurements is provided in Figure 16. As can be seen, the areas with a low probability of occupancy (encoded in light grey) are even lighter, which means that the probability of “emptiness” has been reinforced. The cells with a high probability of occupancy are even darker which means that the probability of occupancy has been reinforced. More noticeable are the cells located between the person and the wall. The depth camera was not able to provide information regarding the presence of an obstacle (in fact “out of range” was returned), leading to the probability 0.5, encoded in grey. After fusion with the ultrasound measurement, these cells are encoded with a lighter grey, because the occupancy probability has decreased. This mechanism is even more reinforced when data from the long-range LiDAR and from the ultra-wideband radar are taken into account in the fusion process. Moreover, this “multi-technology” approach allows detecting obstacles in situations when a single-technology obstacle detection device fails.

## 6. Discussion, and Future Work Directions

The primary goal of INSPEX was to optimize the range sensors embedded in a portable device to detect obstacles in the surrounding of the device. To provide realistic constraints, the smart white cane application was chosen. Table 7 summarizes the characteristics of the four modules optimized in the course of the project. The values that exceed the characteristics are highlighted in bold: the main drawback is the measurement range for the ultra-wideband radar that is halved when compared to its requirements. We explained and quantified this under-performance above. Improvement of this module will require the redesign of the ASIC, which is currently out of the project timeframe.

Power consumption figures correspond to peak measured values. The mean power consumption value is decreased via an ad-hoc power management of the sensing modules designed using formal methods [25]. Remind that we designed each sensor module as a stand-alone one for its partner’s owner to explore other exploitation routes. Consequently, it embeds its own microcontroller with its dedicated firmware (for sensor control, calibration, and data pre-treatment and treatment purposes) and the power supply circuitry. An advanced version of the modules will be less consuming because the firmware of each module will be ported on the INSPEX main computing platform, leading to the remove of the dedicated microcontroller. Moreover, the INSPEX main computing platform delivers the power supply to all the modules, therefore the associated circuitry will be removed from the advanced modules.

**Figure 17:**
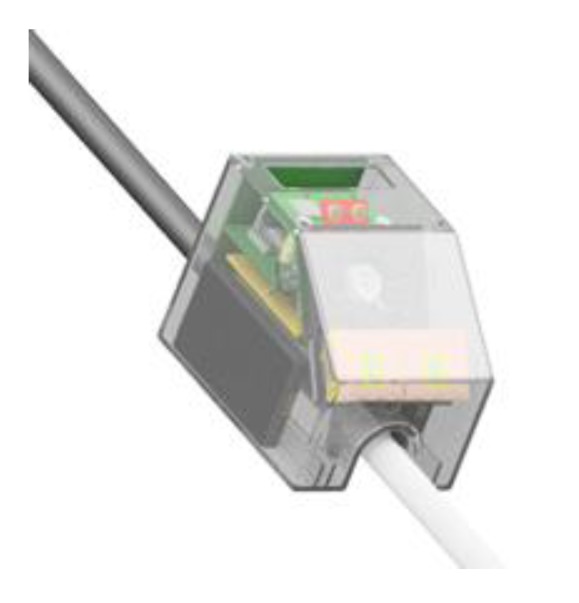
Mockup of the integrated INSPEX device.

The optimized modules have been delivered to the consortium and the final prototype is under assembling. Its mockup is shown in Figure 17.

Information about obstacles entering the user’s “safety cocoon” (see Figure 1) are extracted from the occupancy grid computed by SigmaFusion™ [15]. This information is sent to the user’s smartphone via Bluetooth Low Energy (BLE). 3D spatial sound that provides a virtual image of the obstacle is output to the user with an extra-auricular headset [26].

Tests with the INSPEX integrated portable device will be conducted both in laboratory conditions and with VIB people in realistic environments. The test results are not reported in the present paper whose main objective was to present the optimization work carried out in parallel for each range sensing technology integrated in the portable device.

## Figures and Tables

**Table 1:** Initial system requirements from a sensing perspective for each sensing module.

Sensor Characteristics	Ultrasound	Long-Range LiDAR	Depth Camera	Ultra-Wideband Radar
Measurement range	>1 m	10 m	4 m	4 m
Consumption	<100 mW	<200 mW	<100 mW	<600 mW
Size	<50 cm^3^	<150 cm^3^	<100 cm^3^	<50 cm^3^
Weight	<30 g	<50 g	<50 g	<50 g
Field-of-view	in [20°, 40°]	< 5°	>30°	>50°

**Table 2:** Comparison between the prototype and the optimized modules (ultrasound).

Characteristics	Prototype	Optimized (Designed)	Requirements
Weight (g)	44	25	<30
Dimension (cm^3^)	11 × 4 × 3	5.5 × 4 × 2 = 44	<50
Power (mW)	400	70	<100
Range (m)	<1	>5	>1

**Table 3:** Intensity of the received echo when obstacles are located at 1 m from the ultrasound module.

Shape	Material	Dimension	Intensity (Arbitrary Unit)
Flat surface	wall	-	2450
Cylinder	aluminum	diam. 58 mm	535
Cylinder	steel (painted)	diam. 35 mm	390
Cylinder	plastic	diam. 18 mm	275
Cylinder	plastic	diam. 8 mm	205
Cylinder	wood (painted)	diam. 6 mm	125
Sphere	expanded polystyrene	diam. 244 mm	225
Sphere	expanded polystyrene	diam. 150 mm	125
Sphere	expanded polystyrene	diam. 100 mm	120

**Table 4:** Comparison between the prototype and the optimized module (long-range LiDAR).

Characteristics	Prototype	Optimized (Design)	Optimized (Measured)	Requirements
Weight (g)	465	70	50	50
Dimension (cm^3^)	11.9 × 7.7 × 5.5 = 465.8	6 × 7.7 × 3 = 138.6	4.5 × 5.1 × 3 = 68.85	<150
Power (mW)	1320	500	400	100
Range (m)	25	10	10	10

**Table 5:** Comparison between the prototype and the optimized module (depth camera).

Characteristics	Prototype	Optimized	Requirements
Weight (g)	>100	50	50
Dimension (cm^3^)	9 × 7 × 5 = 315	5 × 4 × 3.8 = 76	<100
Power (mW)	>1000	140	100
Range (m)	>10	4.5	4

**Table 6:** Comparison between the prototype and the optimized module (Ultra-wideband radar).

Characteristics	Prototype	Optimized	Requirements
Weight (g)	200	25	<50
Dimension (cm^3^)	9 × 9 × 3.5	5.6 × 2 × 3 = 33.6	<50
Power (mW)	825	825	<600
Range (m)	3–5	2	4

**Table 7:** Sensing modules requirements vs. characteristics of the optimized modules.

Sensor Characteristics (Required/Achieved)	Ultrasound	Long-Range LiDAR	Depth Camera	Ultra-Wideband Radar
Measurement range (m)	> 1/> 2	10/10	4/4.5	4/**2**
Consumption (mW)	<100/100	<200/**400**	<100/**140**	<600/**825**
Size (cm^3^)	<50/44	<150/138.6	<100/76	<50/33.5
Weight (g)	<30/25	<50/50	<50/50	<50/25
Field-of-view	in [20°, 40°]/25°	<5°/-	>30°/V33.5°× H40°	>50°/V56°× H56°
